# SIRT1 rs3758391 polymorphism and risk of diffuse large B cell lymphoma in a Chinese population

**DOI:** 10.1186/s12935-018-0659-z

**Published:** 2018-10-22

**Authors:** Yutian Kan, Peng Ge, Xinyuan Wang, Gangfeng Xiao, Haifeng Zhao

**Affiliations:** 1 Department of Hematology and Oncology, Tianjin Medical University Cancer Institute and Hospital, National Clinical Research Center for Cancer, Key Laboratory of Cancer Prevention and Therapy, Tianjin Clinical Research Center for Cancer, Tianjin, 300060 People’s Republic of China; 2 Department of Laboratory, Tianjin Medical University Cancer Institute and Hospital, National Clinical Research Center for Cancer, Key Laboratory of Cancer Prevention and Therapy, Tianjin Clinical Research Center for Cancer, Tianjin, People’s Republic of China; 3 Department of Hematology and Oncology, Ningbo No. 2 Hospital, Ningbo Medical University, Ningbo, Zhejiang People’s Republic of China

**Keywords:** DLBCL, Single nucleotide polymorphism, SIRT1

## Abstract

**Background:**

The aim of the study was to explore the association between the SIRT1 single nucleotide polymorphism (SNP) rs3758391 and diffuse large B cell lymphoma (DLBCL) in a Chinese Han population.

**Methods:**

206 patients diagnosed with DLBCL and 219 healthy individuals were recruited in the present study. The genotyping of SIRT1 rs3758391 polymorphism was detected by polymerase chain reaction–restriction fragment length polymorphism. The SIRT1 mRNA expression was detected by the Taqman real-time quantitative PCR.

**Results:**

Our study showed that the genotype TT and allele T frequency were significantly higher in DLBCL patients than that of controls (p = 0.02 and 0.01, respectively). No statistical differences were observed between SIRT1 rs3758391 and clinical characteristics of DLBCL patients. Analysis of the polymorphism revealed an increased risk of DLBCL associated with TC and TT genotype when compared with CC genotype [odds ratio = 2.621 and 3.518, respectively; 95% confidence interval (CI) 1.249–5.501 and 1.675–7.390, respectively; p = 0.011 and 0.001, respectively]. The survival analysis indicated that the patients with C allele had higher overall survival rate than those with genotype TT (p = 0.005). Furthermore, multivariate Cox regression analysis showed that the TT genotype of SIRT1 SNP rs3758391 was an independent poor prognostic factor for DLBCL patients (p = 0.006, HR 1.981, 95% CI 1.215–3.231). The SIRT1 mRNA expression was significantly upregulated in DLBCL patients than that of controls (p < 0.001). In addition, the SIRT1 mRNA expression of TT subgroup was upregulated compared with TC/CC subgroup in DLBCL patients (p < 0.001).

**Conclusion:**

These results suggest that the SIRT1 rs3758391 polymorphism is associated with the risk and survival rate of DLBCL in Chinese Han population.

## Background

Diffuse large B cell lymphoma (DLBCL) is the most common form of B-cell non-Hodgkin lymphoma (NHL). After standard chemoimmunotherapy such as rituximab, cyclophosphamide, doxorubicin, vincristine, and prednisone (R-CHOP), DLBCL is curable in approximately 60% of patients [1]. It can be classified into three subtypes: activated B-cell-like (ABC), germinal-center B-cell-like (GCB) and type 3 according to gene-expression profiling [2, 3]. The molecular subtypes are associated with overall survival and the ABC type indicates a poor prognosis [4]. The International Prognostic Index (IPI) is a powerful prognostic tool for DLBCL patients based on the clinical characteristics. However, the use of IPI predates the use of rituximab and can be enhanced little by novel prognostic markers [5]. Therefore, there is still a lack of robust prognostic tool for DLBCL patients.

Histone acetyltransferases (HATs) and histone deacetylases (HDACs) are crucial modifications that regulate gene transcription [6]. HDACs are related to a number of oncogenes and tumor suppressor genes which lead to aberrant HDAC activities and in return changing gene expression [7, 8]. Sirtuin1 (SIRT1), the yeast silent information regulator 2 (sir2) ortholog, is a nicotinamide adenine dinucleotide (NAD^+^)-dependent histone deacetylase (HADC) [9]. SIRT1 participates in the regulation of diverse cellular processes, including proliferation, differentiation, senescence and apoptosis [10–12]. Knockdown or inhibition of SIRT1 leads to cell cycle arrest and apoptosis in Primary effusion lymphoma (PEL) cells, which is an aggressive B-cell lymphoma associated with Kaposi’s sarcoma-associated herpesvirus infection [13]. Overexpression of SIRT1 is also found in chronic lymphocytic leukemia (CLL) [14]. Nicotinamide can block proliferation and promote apoptosis of chronic lymphocytic leukemia cells with wild-type p53 via the p53/miR-34a/SIRT1 pathway [14].

rs3758391 (T/C), a single nucleotide polymorphism (SNP) of SIRT1 gene promoter, is located at the p53-binding site of the gene. The C variation of the SNP disrupts the p53-binding sequence and affects SIRT1 expression in vitro, which indicates that it may play a significant role in human pathophysiology [15]. In addition, accumulating evidence suggested an important role of the SIRT1 SNP rs3758391 in diverse diseases, including lung cancer, breast cancer, depressive disorders and autoimmune thyroid disease and systemic lupus erythematosus [16–20].

However, the association between SIRT1 rs3758391 polymorphism and the risk of DLBCL in the Chinese Han population has not been fully illustrated. Based on the previous observations, we hypothesize that SIRT1 rs3758391 polymorphism may relate to the susceptibility and prognosis of DLBCL patients. In order to test this hypothesis, we investigated the association between this SNP and DLBCL in Chinese Han people. The results showed that the SIRT1 rs3758391 was associated with the risk and survival rate of DLBCL, indicating its potential use as a biomarker to predict the prognosis of DLBCL patients.

## Methods

### Subjects

Patients pathologically diagnosed with DLBCL and healthy individuals were recruited in the study. 206 peripheral blood specimens were collected from DLBCL patients diagnosed at Tianjin Medical University Cancer Institute and Hospital and Ningbo No. 2 Hospital before initial therapy. 219 healthy people with the same geographical and ethnic background as patients were recruited as controls. The expression of SIRT1 mRNA was determined in 58 patients with DLBCL and 30 patients with reactive hyperplasia lymphoid. The clinical and pathological characteristics of the patients were acquired from medical record review. The general data of the patients enrolled in this study were presented in Table 1. Written informed consent was obtained from each participant, and the entire study was approved by the local Institutional Review Board.

**Table 1:** The clinical characteristics of DLBCL patients

Characteristics	No. (n = 206) n (%)
Gender	
Male	97 (47.09)
Female	109 (52.91)
Age	
≤ 60	145 (70.39)
> 60	61 (29.61)
B symptom	
+	64 (31.07)
−	142 (68.93)
Subtype	
GCB	63 (30.58)
nGCB	143 (69.42)
Ann-arbor stage	
I–II	132 (64.08)
III–IV	74 (35.92)
IPI score	
0–1	106 (51.46)
2–5	100 (48.54)
ECOG	
0–1	193 (93.69)
2–5	13 (6.31)
Extra nodal sites	
+	126 (61.17)
−	80 (38.83)
Bulky tumor (> 10 cm)	
+	48 (23.30)
−	158 (76.70)
Bone marrow involvement	
+	11 (5.34)
−	195 (94.66)
Elevated LDH	
+	85 (41.26)
−	121 (58.73)
Elevated β2-MG	
+	61 (29.61)
−	145 (70.39)
HBV infection	
+	45 (21.84)
−	161 (78.16)
KI-67	
≤ 75%	96 (46.60)
> 75%	110 (53.40)

*LDH* lactate dehydrogenase, *β2*-*MG* β2 macroglobulin, *ECOG* Eastern Cooperative Oncology Group, *IPI* International Prognostic Index

### Extraction of DNA

Peripheral blood samples were collected in vacuum tubes containing 5% EDTA. Genomic DNA was isolated from whole blood with the TIANamp Genomic DNA Kit (TianGen Biotech, Beijing, China) to the manufacturer’s instruction. The genomic DNA was diluted in buffer TE and stored at − 20 °C.

### SIRT1 rs3758391 genotyping by polymerase chain reaction–restriction fragment length polymorphism (PCR–RFLP)

SIRT1 rs3758391 was analyzed through PCR–RFLP, as previously described [19]. The primer for rs3758391 was forward 5′-ACGCAGGTAATTGATGCAGT-3′ and reverse 5′-CGTGAGCTATCTAGCCGTTT-3′. The total reaction system was 50 μl, including 25 μl of Premix Taq (Takara, Dalian, China), 2 μl (0.2 mM) of upstream and downstream primers, respectively, 1 μg of DNA template, and 16 μl of ddH_2_O. PCR conditions were programmed on an ABI 9700 thermal cycler (Applied Biosystems, Carlsbad, CA, USA) as follows: initial denaturation at 94 °C for 5 min, followed by 30 cycles of 94 °C for 30 s, 58 °C for 30 s, 72 °C for 30 s, followed by 72 °C for 7 min. The PCR product was digested by addition of *Nco*I (New England Biolabs, Beverly, MA). The enzyme-digested products were analyzed by gel electrophoresis on 2.5% agarose gels. To confirmed our results, randomly selected amplified DNA samples were examined by direct sequencing method, and the results were 100% concordant.

### RNA isolation

Total RNA was extracted from the DLBCL sample and reactive hyperplasia lymphoid node tissue by Trizol reagent (Invitrogen). The absorbance and purity of RNA were determined by Nanodrop2000 (Thermo Scientific, Wilmington, DE). The ratio of optical density (OD) 260/OD280 between 1.8 and 2.0 was used in subsequent experiments.

### SIRT1 mRNA expression detected by TaqMan real-time PCR

The expression of SIRT1 mRNA was detected by reverse-transcription quantitative PCR (RT-qPCR) experiment on an ABI PRISM-7500 Sequence Detection System (ABI Company, Oyster Bay, NY, USA). The relative expression of glyceraldehyde phosphate dehydrogenase (GAPDH) was used as control, and 2^−ΔΔCt^ indicated the quantification of gene expression. The primer sequences for SIRT1 were: forward 5′-ATGCAAGCTCTAGTGACTGGACT-3′, reverse 5′-CTCAGGTGGAGGTATTGTTTCC-3′. The primer sequences for GAPDH were: forward 5′-CCACATCGCTCAGACACCAT-3′, reverse 5′-CCAGGCGCCCAATACG-3′.

### Statistical analysis

All statistical analyses were performed using IBM SPSS Statistics version 20.0 (SPSS Inc., Chicago, IL, USA). Hardy–Weinberg equilibrium Test was estimated by a goodness-of-fit χ^2^ test. The allelic frequency and genotype distributions between patients and controls were compared using Chi-square tests. The association between polymorphism and the clinical characteristics of DLBCL patients were analyzed by χ^2^ test. The association between polymorphism and the risk for DLBCL was calculated by unconditional logistic regression. The survival curves were constructed by Kaplan–Meier method and compared between groups by the log-rank tests. Association between the genotypes of patients and the overall survival was estimated by univariate Cox regression analysis. The differences in the levels of SIRT1 mRNA expression were analyzed by Student’s t-test. All tests were two-sided, and p < 0.05 was considered to indicate statistically significant.

## Results

### Association between the SIRT1 rs3758391 and the risk of DLBCL

**Fig. 1:**
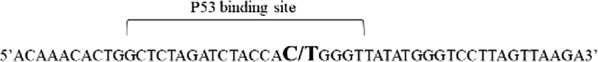
The sequence of rs3758391 polymorphism in the human SIRT1 promoter and the location of the p53-binding site [15]

To determine the association between SIRT1 rs3758391 and DLBCL, a case–control study including 206 DLBCL patients and 219 control subjects was performed. The sequence of rs3758391 polymorphism and the location of the p53-binding site were shown in Fig. 1. The genotype distributions in both groups agreed with the predicted distribution under the Hardy–Weinberg equilibrium (χ^2^ = 2.473, p = 0.290; χ^2^ = 0.070, p = 0.965, respectively). No significant difference between patients and controls was observed regarding gender and age (χ^2^ = 0.011, p = 0.916; χ^2^ = 0.680, 0.410, respectively), indicating that the frequency matching is sufficient. The genotype and allele frequency were significantly different between patients and controls for SIRT1 rs3758391 (p = 0.02, 0.01, respectively) as shown in Table 2, suggesting that the difference was statistically significant. However, the percentage of TC genotypes in the case group was only slightly lower than that of the control group (44.17% versus 46.12%). No statistical differences were observed between SIRT1 rs3758391 and clinical characteristics of DLBCL patients as shown in Table 3. Further investigation showed that the TC and TT genotype were statistically significantly associated with increased risk of DLBCL [odds ratio (OR) = 2.621 and 3.518, respectively; 95% confidence interval (CI) 1.249–5.501 and 1.675–7.390, respectively; p = 0.011 and 0.001, respectively, shown in Table 4].

**Table 2:** SIRT1 rs3758391 polymorphism in DLBCL patients and the controls

	Control (n = 219) n (%)	DLBCL patients (n = 206) n (%)	p
Genotype frequency			
TT	86 (39.27)	104 (50.49)	0.002
TC	101 (46.12)	91 (44.17)	
CC	32 (14.61)	11 (5.34)	
Allele frequency			
T	273 (62.33)	299 (72.57)	0.001
C	165 (37.67)	113 (27.43)

**Table 3:** Association between SIRT1 rs3758391 and clinical characteristics of DLBCL patients

Variables	No.	Genotype		p
	(n = 206) n (%)	CC + CT n (%)	TT n (%)	
Gender				
Male	97 (47.09)	52 (25.24)	45 (21.84)	0.268
Female	109 (52.91)	50 (24.27)	59 (28.64)	
Age				
≤ 60	145 (70.39)	76 (36.89)	69 (33.50)	0.199
> 60	61 (29.61)	26 (12.62)	35 (16.99)	
B symptom				
+	64 (31.07)	28 (13.59)	36 (17.48)	0.267
−	142 (68.93)	74 (35.92)	68 (33.01)	
Subtype				
GCB	63 (30.58)	32 (15.53)	31 (15.05)	0.807
nGCB	143 (69.42)	70 (33.98)	73 (35.44)	
Ann-arbor stage				
I–II	132 (64.08)	61 (29.61)	71 (34.47)	0.205
III–IV	74 (35.92)	41 (19.90)	33 (16.02)	
IPI score				
0–1	106 (51.46)	58 (28.16)	48 (23.30)	0.124
2–5	100 (48.54)	44 (21.36)	56 (27.18)	
ECOG				
0–1	193 (93.69)	97 (47.09)	96 (46.60)	0.410
2–5	13 (6.31)	5 (2.43)	8 (3.88)	
Extra nodal sites				
+	126 (61.17)	67 (32.52)	59 (28.64)	0.187
−	80 (38.83)	35 (16.99)	45 (21.84)	
Bulky tumor (> 10 cm)				
+	48 (23.30)	26 (12.62)	22 (10.68)	0.462
−	158 (76.70)	76 (36.89)	82 (39.81)	
Bone marrow involvement				
+	11 (5.34)	5 (2.43)	6 (2.91)	0.782
−	195 (94.66)	97 (47.09)	98 (47.57)	
Elevated LDH				
+	85 (41.26)	46 (22.33)	39 (18.93)	0.268
−	121 (58.73)	56 (27.18)	65 (31.55)	
Elevated β2-MG				
+	61 (29.61)	28 (13.59)	33 (16.02)	0.501
−	145 (70.39)	74 (35.92)	71 (34.47)	
HBV infection				
+	45 (21.84)	23 (11.17)	22 (10.68)	0.809
−	161 (78.16)	79 (38.35)	82 (39.81)	
KI-67				
≤ 75%	96 (46.60)	42 (20.39)	54 (26.21)	0.122
> 75%	110 (53.40)	60 (29.13)	50 (24.27)	

*LDH* lactate dehydrogenase, *β2*-*MG* β2 macroglobulin, *ECOG* Eastern Cooperative Oncology Group, *IPI* International Prognostic Index

**Table 4:** Association between SIRT1 rs3758391 polymorphism and the risk of DLBCL

	Control (n = 219) n (%)	DLBCL patients (n = 206) n (%)	OR (95% CI)	p
Genotype				
CC	32 (14.16)	11 (5.43)	1 (reference)	
TC	101 (46.12)	91 (44.17)	2.621 (1.249–5.501)	0.011
TT	86 (39.72)	104 (50.49)	3.518 (1.675–7.390)	0.001

*OR* odds ratio, *95% CI* 95% confidence interval

### Association between the SIRT1 rs3758391 and the survival rate of DLBCL

**Fig. 2:**
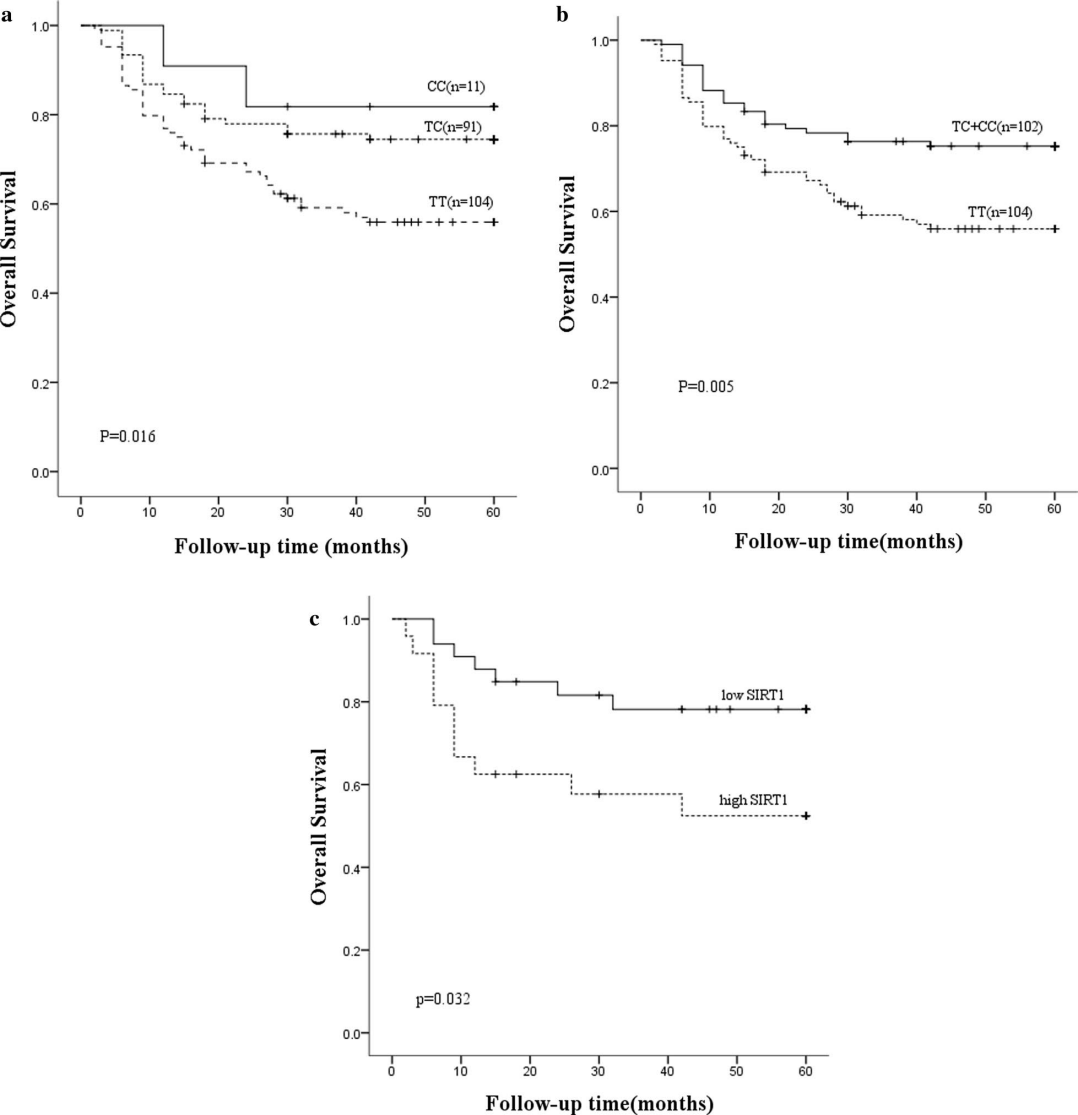
Kaplan–Meier analyses of overall survival for DLBCL patients. **a** Overall survival for 206 DLBCL patients according to genotype of SIRT1 rs3758391. Comparison among CC, TC and TT genotypes (p = 0.016). **b** Overall survival for 206 DLBCL patients according to genotype of SIRT1 rs3758391. Comparison between TT genotype and combined CC/TC genotypes (p = 0.005). **c** Overall survival for 58 DLBCL patients according to SIRT1 mRNA level. The overall survival of the low-SIRT1 subgroup was significantly higher than that of the high-SIRT1 subgroup (p = 0.032). Log-rank p values were indicated. Tick marks represent censored data

All 206 DLBCL patients were included in the survival analysis. The effect of SIRT1 rs3758391 on the overall survival rate of DLBCL patients was investigated using Kaplan–Meier curve and log-rank test. The subjects with CC genotype in our study population were small (n = 115.34%, shown in Table 2). Therefore, the CC genotype was combined with the TC genotype for survival analysis of DLBCL. As expected, the survival analysis showed that the patients with C allele had higher 5-year overall survival rate than those with genotype TT (p = 0.005, Fig. 2). Furthermore, multivariate Cox regression analysis indicated that patients with C allele displayed better survival rate compared with those with TT genotype (p = 0.006, HR 1.981, 95% CI 1.215–3.231).

### Association between the SIRT1 mRNA level and the survival of DLBCL

Further investigation focused on the association between the SIRT1 mRNA level and the survival rate of DLBCL. 58 DLBCL patients were included in the analysis. According to the SIRT1 mRNA levels, the patients were divided into high-SIRT1 group (above the median) and low-SIRT1 group (below the median). The survival analysis showed that the patients with lower SIRT1 mRNA level had higher 5-year overall survival rate than those with high SIRT1 mRNA level (p = 0.032, Fig. 2c). Multivariate Cox regression analysis indicated that patients with low SIRT1 level displayed better survival rate compared with those high SIRT1 level (p = 0.043, HR 2.661, 95% CI 1.029–6.880).

### Association between the SIRT1 rs3758391 and SIRT1 mRNA expression levels

**Fig. 3:**
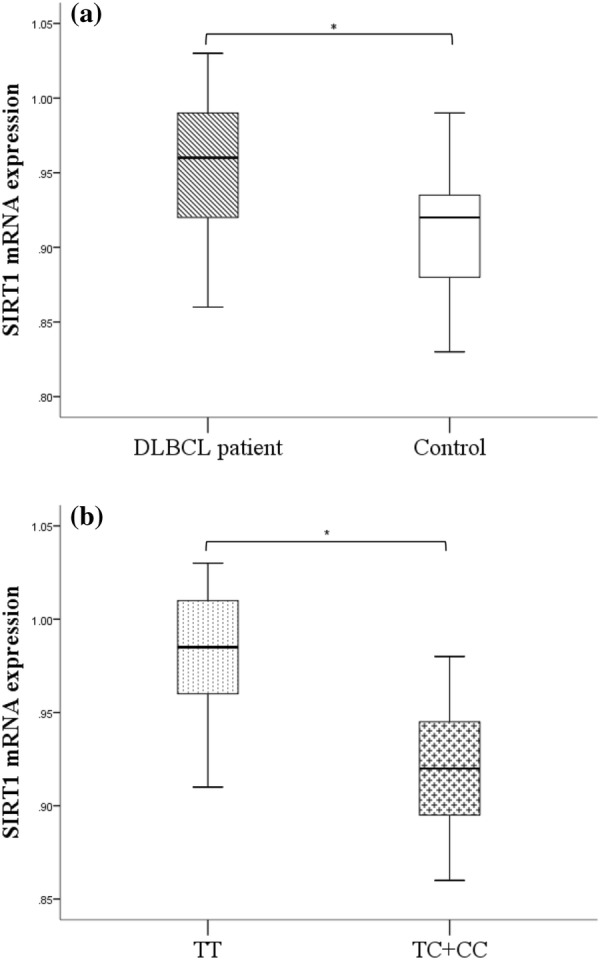
SIRT1 mRNA expression DLBCL patients and reactive hyperplasia lymphoid patients as controls. The SIRT1 mRNA expression was detected by the TaqMan RT-PCR and calculated by Student’s t-test. **a** SIRT1 mRNA expression in DLBCL patients and was notably upregulated than that of controls (p < 0.001). **b** SIRT1 mRNA expression in DLBCL patients with TT genotype was significantly upregulated than that with TC/CC genotype (p < 0.001). *p < 0.001

The SIRT1 mRNA expression of 58 DLBCL patients and 30 controls were detected by Student’s t-test. It was shown that the SIRT1 mRNA expression was significantly upregulated in DLBCL patients than that of controls (p < 0.001, Fig. 3a). Subsequently, we analyzed the SIRT1 mRNA expression between different genotypes in DLBCL patients. The SIRT1 mRNA expression of TT subgroup was upregulated compared with TC/CC subgroup in DLBCL patients (p < 0.001, Fig. 3b).

## Discussion

The balance between the complex activities of histone acetyltransferases and histone deacetylases is associated with gene expression [21, 22]. In addition to histones, other substrates can also be deacetylated by HDACs, such as p53 [23]. SIRT1 is a highly conserved protein deacetylase. Accumulating evidence indicates that SIRT1 is a key regulator of diverse life events, including life span extension, inflammation, and cancer [24]. In cancer, the role of SIRT1 is controversial since it can function as both a tumor promoter and tumor suppressor [25–27]. SIRT1 can deacetylate histone H4 lysine 16 (H4K16) [28], whose hypoacetylation is a hallmark of human cancer [29]. SIRT1 can also deacetylate p53 at lysine 382 and decrease its transcription, resulting in an obstruction of p53-dependent apoptosis in response to DNA damage signals [30, 31].

SIRT1 rs3758391 (T/C) was located at the p53-binding site of the SIRT1 gene [15]. In the present study, we explored the relationship between the polymorphism and DLBCL in Chinese Han people. Unfortunately, no significant differences were observed between the genotypes and clinical characteristics such as age, sexual, subtype and clinical stage. We found that the T allele carriers were statistically significantly associated with the increased risk of DLBCL. SIRT1 mRNA expression was upregulated in DLBCL patients with TT genotype. Moreover, patients with TT genotypes displayed worse survival. These results suggest that the SIRT1 rs3758391 may be used as a biomarker for the prediction of susceptibility and survival of DLBCL.

SIRT1 rs3758391 was reported to be common with an overall average heterozygosity frequency of 50% in the HapMap Project. In white Americans of Northern and Western European ancestry and African–Americans, the frequency of T allele was 25–35%, whereas in Japanese and Chinese, the frequency was 80–90% [15]. However, our data showed a minor difference in distribution of allele frequency compared to those in Han Chinese from HapMap data. Our analysis indicated that the frequency of allele T in DLBCL patients and controls was 72.57% and 62.33%, respectively. This difference in allele frequency may due to the sample size.

In summary, our results suggest that the SIRT1 rs3758391 is associated with the risk and survival rate of DLBCL in Chinese Han people. The DLBCL patients with genotype CC of SIRT1 SNP rs3758391 show better survival rate than those with allele T. Clearly, additional investigations are necessary to confirm our findings. Therefore, SIRT1 SNP rs3758391 could be considered as an independent biomarker to predict the prognosis in DLBCL patients.

## Conclusion

This study reveals a key role of SIRT1 rs3758391 in the risk and survival rate of DLBCL in Chinese Han people. Therefore, SIRT1 could be a potential biomarker and therapeutic target in the prognosis and treatment of DLBCL.

## Abbreviations

DLBCL: diffuse large B cell lymphoma
NHL: non-Hodgkin’s lymphoma
GCB: germinal center B-cell
ABC: activated B-cell
IPI: International Prognostic Index
HAT: histone acetyltransferase
HDAC: histone deacetylase
SNP: single nucleotide polymorphism
ECOG: Eastern Cooperative Oncology Group
LDH: lactate dehydrogenase
β2-MG: β2 macroglobulin
HBV: hepatitis-B virus

## References

[1] Coiffier B, Lepage E, Briere J, Herbrecht R, Tilly H, Bouabdallah R, Morel P, Van Den Neste E, Salles G, Gaulard P (2002). CHOP chemotherapy plus rituximab compared with CHOP alone in elderly patients with diffuse large-B-cell lymphoma. N Engl J Med.

[2] Alizadeh AA, Eisen MB, Davis RE, Ma C, Lossos IS, Rosenwald A, Boldrick JC, Sabet H, Tran T, Yu X (2000). Distinct types of diffuse large B-cell lymphoma identified by gene expression profiling. Nature.

[3] Rosenwald A, Wright G, Chan WC, Connors JM, Campo E, Fisher RI, Gascoyne RD, Muller-Hermelink HK, Smeland EB, Giltnane JM (2002). The use of molecular profiling to predict survival after chemotherapy for diffuse large-B-cell lymphoma. N Engl J Med.

[4] Morin RD, Assouline S, Alcaide M, Mohajeri A, Johnston RL, Chong L, Grewal J, Yu S, Fornika D, Bushell K (2016). Genetic landscapes of relapsed and refractory diffuse large B-cell lymphomas. Clin Cancer Res.

[5] Zhou Z, Sehn LH, Rademaker AW, Gordon LI, Lacasce AS, Crosby-Thompson A, Vanderplas A, Zelenetz AD, Abel GA, Rodriguez MA (2014). An enhanced International Prognostic Index (NCCN-IPI) for patients with diffuse large B-cell lymphoma treated in the rituximab era. Blood.

[6] Yang XJ, Seto E (2007). HATs and HDACs: from structure, function and regulation to novel strategies for therapy and prevention. Oncogene.

[7] de Ruijter AJ, van Gennip AH, Caron HN, Kemp S, van Kuilenburg AB (2003). Histone deacetylases (HDACs): characterization of the classical HDAC family. Biochem J.

[8] Cress WD, Seto E (2000). Histone deacetylases, transcriptional control, and cancer. J Cell Physiol.

[9] Lin Z, Fang D (2013). The roles of SIRT1 in cancer. Genes Cancer.

[10] Haigis MC, Guarente LP (2006). Mammalian sirtuins—emerging roles in physiology, aging, and calorie restriction. Genes Dev.

[11] Haigis MC, Sinclair DA (2010). Mammalian sirtuins: biological insights and disease relevance. Annu Rev Pathol.

[12] Brooks CL, Gu W (2009). How does SIRT1 affect metabolism, senescence and cancer?. Nat Rev Cancer.

[13] He M, Tan B, Vasan K, Yuan H, Cheng F, Ramos da Silva S, Lu C, Gao SJ (2017). SIRT1 and AMPK pathways are essential for the proliferation and survival of primary effusion lymphoma cells. J Pathol.

[14] Audrito V, Vaisitti T, Rossi D, Gottardi D, D’Arena G, Laurenti L, Gaidano G, Malavasi F, Deaglio S (2011). Nicotinamide blocks proliferation and induces apoptosis of chronic lymphocytic leukemia cells through activation of the p53/miR-34a/SIRT1 tumor suppressor network. Cancer Res.

[15] Naqvi A, Hoffman TA, DeRicco J, Kumar A, Kim CS, Jung SB, Yamamori T, Kim YR, Mehdi F, Kumar S (2010). A single-nucleotide variation in a p53-binding site affects nutrient-sensitive human SIRT1 expression. Hum Mol Genet.

[16] Lv Y, Lin S, Peng F (2017). SIRT1 gene polymorphisms and risk of lung cancer. Cancer Manag Res.

[17] Rizk SM, Shahin NN, Shaker OG (2016). Association between SIRT1 gene polymorphisms and breast cancer in Egyptians. PLoS ONE.

[18] Kovanen L, Donner K, Partonen T (2015). SIRT1 polymorphisms associate with seasonal weight variation, depressive disorders, and diastolic blood pressure in the general population. PLoS ONE.

[19] Sarumaru M, Watanabe M, Inoue N, Hisamoto Y, Morita E, Arakawa Y, Hidaka Y, Iwatani Y (2016). Association between functional SIRT1 polymorphisms and the clinical characteristics of patients with autoimmune thyroid disease. Autoimmunity.

[20] Consiglio CR, Juliana da Silveira S, Monticielo OA, Xavier RM, Brenol JC, Chies JA (2014). SIRT1 promoter polymorphisms as clinical modifiers on systemic lupus erythematosus. Mol Biol Rep.

[21] Wade PA (2001). Transcriptional control at regulatory checkpoints by histone deacetylases: molecular connections between cancer and chromatin. Hum Mol Genet.

[22] Forsberg EC, Bresnick EH (2001). Histone acetylation beyond promoters: long-range acetylation patterns in the chromatin world. BioEssays.

[23] Juan LJ, Shia WJ, Chen MH, Yang WM, Seto E, Lin YS, Wu CW (2000). Histone deacetylases specifically down-regulate p53-dependent gene activation. J Biol Chem.

[24] Yang H, Bi Y, Xue L, Wang J, Lu Y, Zhang Z, Chen X, Chu Y, Yang R, Wang R (2015). Multifaceted modulation of SIRT1 in cancer and inflammation. Crit Rev Oncog.

[25] Karbasforooshan H, Roohbakhsh A, Karimi G (2018). SIRT1 and microRNAs: the role in breast, lung and prostate cancers. Exp Cell Res.

[26] Portmann S, Fahrner R, Lechleiter A, Keogh A, Overney S, Laemmle A, Mikami K, Montani M, Tschan MP, Candinas D (2013). Antitumor effect of SIRT1 inhibition in human HCC tumor models in vitro and in vivo. Mol Cancer Ther.

[27] Yu Y, Liu Y, Zong C, Yu Q, Yang X, Liang L, Ye F, Nong L, Jia Y, Lu Y (2016). Mesenchymal stem cells with Sirt1 overexpression suppress breast tumor growth via chemokine-dependent natural killer cells recruitment. Sci Rep.

[28] Imai S, Armstrong CM, Kaeberlein M, Guarente L (2000). Transcriptional silencing and longevity protein Sir2 is an NAD-dependent histone deacetylase. Nature.

[29] Fraga MF, Ballestar E, Villar-Garea A, Boix-Chornet M, Espada J, Schotta G, Bonaldi T, Haydon C, Ropero S, Petrie K (2005). Loss of acetylation at Lys16 and trimethylation at Lys20 of histone H4 is a common hallmark of human cancer. Nat Genet.

[30] Luo J, Nikolaev AY, Imai S, Chen D, Su F, Shiloh A, Guarente L, Gu W (2001). Negative control of p53 by Sir2alpha promotes cell survival under stress. Cell.

[31] Vaziri H, Dessain SK, Ng Eaton E, Imai SI, Frye RA, Pandita TK, Guarente L, Weinberg RA (2001). hSIR2(SIRT1) functions as an NAD-dependent p53 deacetylase. Cell.

